# Novel urinary biomarkers to differentiate AKI etiologies and predict mortality in decompensated cirrhosis patients: a prospective cohort study

**DOI:** 10.1186/s12876-025-04462-1

**Published:** 2025-12-07

**Authors:** Chiranjita Phukan, Anjan Jyoti Talukdar, Sangitanjan Dutta, Saptadweep Saha, Monalisha Saikia Borah, Pinku Mani Talukdar, Bhaskar Jyoti Sarma, Achyut Chandra Baishya, Saswati Sanyal Choudhury, Anuradha Deuri

**Affiliations:** 1 Nalbari Medical College and Hospital, Dakhingaon, Nonoi, Nalbari, Assam 781350 India; 2 Gauhati Medical College and Hospital, Bhangagarh, Guwahati, Kamrup (M), Assam 781032 India; 3 Multidisciplinary Research Unit (MRU), Gauhati Medical College and Hospital, Guwahati, Assam India; 4 Social and Preventive Medicine and Principal cum Chief Superintendent, Gauhati Medical College and Hospital, Guwahati, Assam India; 5 Obstetrics and Gynecology and Nodal Officer of Multidisciplinary Research Unit (MRU), Gauhati Medical College and Hospital, Guwahati, Assam India

**Keywords:** Decompensated cirrhosis, Acute tubular necrosis, Hepatorenal syndrome, Pre-renal azotemia, Acute kidney injury, NGAL (lipocalin 2), IL-18 (interleukin 18), KIM-1 (kidney injury molecule 1)

## Abstract

**Introduction:**

The differentiation of causes of Acute Kidney Injury (AKI) in decompensated chronic liver disease (DCLD) presents a diagnostic challenge. This study aims to evaluate the diagnostic and prognostic utility of Neutrophil gelatinase-associated lipocalin (NGAL), Kidney injury molecule-1 (KIM-1), and interleukin-18 (IL-18) in cirrhotic patients with AKI.

**Methods:**

This prospective cohort study was conducted at a tertiary care hospital in northeast India. Patients (*n* = 100) were divided into cases comprising decompensated cirrhosis with AKI (*n* = 50) and controls comprising decompensated cirrhosis without AKI (*n* = 50). Cases included patients with acute tubular necrosis (ATN) (*n* = 10), hepatorenal syndrome (HRS) (*n* = 18), and prerenal azotemia (PRA) (*n* = 22). Urinary NGAL, IL-18 and KIM-1 were analyzed statistically to distinguish the causes of AKI and predict 90-day mortality.

**Results:**

NGAL [cutoff: 339.6 pg/mL, sensitivity: 80%, specificity: 83.3%, Area Under the Receiver Operating Characteristic Curve (AUROC): 0.867] and IL-18 (cutoff: 67.75 pg/mL, sensitivity: 70%, specificity: 88.9%, AUROC: 0.839) differentiated ATN from HRS. NGAL (cutoff: 358.15 pg/mL, AUROC: 0.878) and IL-18 (67.75 pg/mL, AUROC: 0.885) distinguished ATN from non-ATN cases. IL-18 (cutoff: 30.95 pg/mL, AUROC: 0.732) effectively differentiated HRS from PRA cases. KIM-1 had a modest diagnostic performance comparable to serum creatinine. NGAL significantly predicted 90-day mortality (Adjusted Odds Ratio: 1.024; *p* = 0.014).

**Conclusion:**

Both IL-18 and NGAL can assist clinicians in differentiating the causes of AKI while improving the prognostic ability of existing scores in DCLD patients.

**Supplementary Information:**

The online version contains supplementary material available at 10.1186/s12876-025-04462-1.

## Introduction

Acute kidney injury (AKI) is a catastrophic but routine complication of cirrhosis occurring in up to 29% of individuals with the disease. AKI in cirrhosis independently carries a six-fold higher risk of in-hospital mortality compared to cirrhosis without AKI [1]. Hepatorenal syndrome (HRS) and acute tubular necrosis (ATN) are among the most frequent causes of AKI in cirrhosis [2]. Since their prognoses and treatment are quite different, it is essential to distinguish between them. Serum creatinine, although widely used as a marker of kidney function, is an unreliable indicator in cirrhosis due to multiple confounding factors, including reduced creatinine production secondary to hepatic dysfunction, increased tubular secretion, decreased muscle mass, and hyperbilirubinemia that interferes with the creatinine assay. Moreover, creatinine does not reveal the underlying cause of AKI [3]. The revised guidelines for diagnosis of HRS recommended by the International Club of Ascites (ICA) have not translated into better prognosis, especially in critically ill patients with cirrhosis [4].

Currently, clinicians evaluate patient history and assess treatment response to diagnose the cause of AKI [3]. The HRS treatment involves volume expansion and administration of vasoconstrictors such as terlipressin, which counters splanchnic vasodilation to increase effective circulating volume and renal perfusion [5]. In contrast, ATN management involves supportive treatment like removing nephrotoxic insults and restricting fluid intake [6]. Absence of reliable biomarkers to distinguish between the two compels physicians to use a trial-and-error approach, which may delay appropriate treatment. For instance, patients with ATN who receive vasoconstrictors derive no benefit and may suffer serious adverse effects such as respiratory failure. At the same time, those with HRS may experience treatment delays, increasing mortality risk [7, 8].

Neutrophil gelatinase-associated lipocalin (NGAL), kidney injury molecule-1 (KIM-1), and interleukin-18 (IL-18) are among the most promising candidates for distinguishing AKI subtypes. These biomarkers may also facilitate earlier detection of AKI and help resolve the diagnostic uncertainty between HRS and ATN [9]. Emerging evidence suggests that these biomarkers could play a role analogous to troponins in the early diagnosis of myocardial infarction [10].

NGAL is a small protein released from injured tubular cells of the kidney in response to injury due to ischemia, toxins, etc [11]. KIM-1 is a transmembrane protein in proximal tubules that is highly expressed in response to kidney damage, indicating structural injury in the kidney [12]. Interleukin 18 (IL-18) is a pro-inflammatory cytokine released by tubular cells due to renal inflammation [13].

The US Food and Drug Administration (FDA) has already approved urinary NGAL for early identification of pediatric patients at risk of developing AKI, thus validating the promise shown by these biomarkers [14]. Since studies have largely focused on the Western population, our study analyzes the diagnostic and prognostic utility of NGAL, KIM-1, and IL-18 in cirrhotic individuals with AKI in an Indian tertiary care setting. Our study is among the first to investigate all three biomarkers in a cirrhosis-specific cohort for both diagnostic and prognostic utility, thus addressing a vital research gap.

## Materials and methodology

### Study design and setting

This prospective cohort study was conducted from May 2023 to March 2025 at Gauhati Medical College and Hospital, Guwahati, India. Participants were enrolled from the Department of Internal Medicine after obtaining ethical clearance from the Institutional Ethics Committee.

### Study population and eligibility criteria

A total of 100 consecutive participants, consisting of 50 cases and 50 controls, were enrolled in the study. Because this is among the first studies to characterize these biomarkers in Indian patients with decompensated chronic liver disease (DCLD-AKI), we adopted a pilot-study framework. Pilot guidelines recommend 30–50 participants per arm to obtain stable estimates of variance and effect size for subsequent sample size calculation in future multicenter studies. Although originally proposed for randomized control trials, they can be extended to a pilot framework for diagnostic studies [15]. Adult patients (>16 years of age) with decompensated cirrhosis and azotemia were identified as cases, whereas individuals with decompensated cirrhosis without any evidence of azotemia were listed as controls. Exclusion criteria comprised: patients with HRS-NAKI (formerly HRS Type II), prior kidney or liver transplant, previously receiving renal replacement therapy, urinary tract infection (UTI), and known renal diseases such as glomerulonephritis or obstructive uropathy, congestive heart failure with a New York Heart Association Grade >2, hepatocellular carcinoma or extrahepatic malignancies, and chronic obstructive pulmonary disease with a grade >2 as per Global Initiative for chronic obstructive disease; pregnant patients and patients not giving consent for the study. The diseases mentioned in the exclusion criteria above were chosen to avoid confounding effects as NGAL, IL-18, KIM-1 are known to be raised in these conditions [11–13].

### Definitions

Cirrhosis was diagnosed based on established clinical guidelines, incorporating clinical, laboratory, and imaging findings. Decompensated cirrhosis was defined by the presence of one or more of the following: jaundice, ascites, variceal bleeding, or hepatic encephalopathy [16]. AKI was defined as per the standard KDIGO (Kidney Disease: Improving Global Outcomes) criteria of an increase in serum creatinine by ≥ 0.3 mg/dl within 48 h, or a rise in serum creatinine to >1.5 times baseline, or urine output < 0.5 mL/kg/h. HRS was diagnosed based on ICA criteria, which include the absence of shock and nephrotoxic drug use, failure to improve kidney function despite diuretic withdrawal and albumin volume expansion within 48 h, among others [3]. ATN was diagnosed based on a history of nephrotoxin exposure and/or hypovolemia, failure to respond to volume administration, descriptive medical records, and a specialist’s evaluation. Due to the inherent sodium retention in cirrhosis, urinary sodium alone was not relied upon for diagnosing ATN, as FENa (Fractional excretion of sodium) can be < 1% even in ATN [17].

### Data collection

Clinical, demographic, and laboratory parameters were recorded in a pre-designed questionnaire which is provided in Supplementary Material 1. Urine samples for analysis were collected, and the MELD-Na and CTP (Child-Turcotte-Pugh) scores were calculated within 24 h of hospital admission. The patients were followed up for 90 days from the day of hospital admission.

### Primary predictors and outcomes

This study aimed to evaluate urinary concentrations of NGAL, IL-18, and KIM-1 in ATN as compared to other causes of AKI. The secondary objective was to assess the ability of biomarkers to predict 90-day mortality in DCLD-AKI patients. 10 mL early-morning, mid-stream urine was collected aseptically from the participants and centrifuged at 2000 g for 20 min; the supernatant was stored at −80 degrees Celsius and retained for analysis. The urinary concentrations of NGAL, KIM − 1, and IL-18 were measured by commercially available ELISA kits (CLOUD-CLONE CORP., Houston, U.S.A) as per the manufacturer’s instructions by laboratory personnel blinded to patient information. Detection range of NGAL, IL-18, and KIM-1 was 156–10000 pg/ml, 15.6–1000 pg/ml, and 0.078–5.078 ng/ml respectively. The inter assay and intra-assay variability was less than 12% and less than 10% respectively [expressed in terms of coefficient of variation (C.V)].

### Statistical analysis

All statistical analyses were performed by using SPSS Version 26. Continuous variables were expressed as medians with interquartile ranges and categorical variables were expressed as frequencies and percentages. The Mann-Whitney U test was used to compare nonparametric continuous data. An independent samples t-test was used to compare parametric data. Normality was assessed using the Shapiro-Wilk test. Given our small sample size, Fisher’s exact test was used for comparing categorical variables. Variables that significantly predicted mortality in univariate analysis were included in various multivariate logistic regression models for predicting short-term mortality. Adjusted odds ratio (AOR) with a 95% confidence interval was calculated for predictors of mortality. Receiver Operating Characteristic (ROC) analysis was done, and AUROC (Area Under ROC) values, sensitivity, specificity, positive predictive value, and negative predictive value of each biomarker in determining the type of AKI, as well as predicting 90-day mortality, were obtained. Optimal cutoffs were determined using Youden’s Index. Pre-test and post-test probabilities of NGAL in diagnosing ATN were calculated. A *p*-value less than 0.05(two-tailed) was considered significant.

## Results

Among the 50 patients in our case group, 10 (20%) were diagnosed with ATN, whereas there were 18 (36%) and 22 (44%) cases of HRS and PRA, respectively. Alcohol was the most common cause of decompensated cirrhosis in our study, accounting for 74% and 72% of controls and cases, respectively. The study population was largely male, comprising 78% of controls and 94% of cases (Table 1).

**Table 1:** Comparison of baseline characteristics, etiology of cirrhosis, comorbidities, laboratory parameters, and novel urinary biomarkers between various patient groups

	Controls (*n* = 50)	Cases (*n* = 50)	ATN (*n* = 10)	HRS (*n* = 18)	PRA (*n* = 22)	*P* value (cases vs. controls)	*P* value (ATN vs. HRS)	*P* value (HRS vs. PRA)	*P* value (ATN vs. PRA)
Age (years) [median (interquartile range)]	41 [17.25]	42.5 [20.25]	40.5 [19.25]	37.5 [18.5]	45 [21]	0.967	0.980	0.600	0.562
Sex									
Male	39 (78%)	47 (94%)	10 (100%)	16 (88.8%)	21 (95.4%)	**0.047**	0.523	0.579	0.999
Female	11 (22%)	3 (6%)	0	2 (11.1%)	1 (4.5%)	-	-	-	-
Etiology of cirrhosis									
Alcoholic	37 (74%)	36 (72%)	8 (90%)	14 (88.8%)	14 (63.63%)	0.999	0.999	0.490	0.439
MASH	7 (14%)	8 (4%)	0	4 (11.1%)	4 (18.18%)	-	-	-	-
Viral (Hepatitis B/Hepatitis C)	4 (8%)	2 (4%)	1 (10%)	0	1 (4.5%)	-	-	-	-
Others (Autoimmune, Wilson’s disease, etc.)	2 (4%)	4 (8%)	1 (10%)	0	3(13.63%)	-	-	-	-
Comorbidities									
Diabetes	14 (28%)	11(22%)	3 (30%)	5 (27.7%)	3 (13.6%)	0.644	0.999	0.429	0.346
Hypertension	9 (18%)	7(14%)	1(10%)	2 (11.1%)	4 (18.2%)	0.758	0.999	0.672	0.999
Signs of decompensated liver disease									
Ascites	38 (76%)	41 (82%)	8 (80%)	16 (88.8%)	17 (77.3%)	0.624	0.601	0.427	0.999
Hepatic encephalopathy	8 (16%)	13 (26%)	3 (30%)	7 (38.8%)	3 (13.6%)	0.326	0.702	0.140	0.346
SBP	9 (18%)	13 (26%)	4 (40%)	5 (27.7%)	4 (18.18%)	0.469	0.677	0.705	0.218
UGI bleed	10 (20%)	11 (22%)	2 (20%)	4 (22.2%)	5 (22.7%)	0.999	0.999	0.999	0.999
Laboratory parameters									
Serum creatinine (mg/dL)	1.15 (1.17)	1.85 (1.00)	2.3 (3.03)	2.05 (1.2)	1.55 (0.52)	**< 0.001**	0.245	0.180	**0.001**
Serum bilirubin (mg/dL)	2.4 (1.53)	3.2 (4.08)	12.95 (19.27)	3.95 (9.98)	2.25 (1.53)	**0.016**	**0.035**	**0.008**	**0.001**
INR	1.4 (0.63)	1.4 (0.5)	1.45 (0.55)	1.65 (0.77)	1.2 (0.5)	0.416	0.356	**< 0.001**	0.052
Serum sodium (mEq/L)	133.5 (9.0)	132 (4.0)	131 (5.0)	131 (4.75)	132 (0.35)	0.065	0.759	0.581	0.483
Albumin (g/dL)	2.90 (0.66)	2.75 (0.7)	2.7 (1.0)	2.7 (0.7)	2.8 (0.88)	0.676	0.411	0.233	0.513
Duration of hospital stay (days)	6 (5.0)	10.5 (7.25	11.5 (3.75	13.5 (7.25)	6 (4.5)	**0.003**	0.259	**0.043**	0.260
Total leukocyte (counts per µL)	7635 (4067.5)	9150 (9862.5)	14,015 (11717.5)	9735 (8725.0)	7350 (10547.5)	**0.033**	0.436	0.132	0.077
Clinical prognostic scores									
MELD-Na	20 (7.25)	24 (7.75)	30.5 (11.25)	26 (7.25)	20 (4.0)	**0.001**	**0.039**	**< 0.001**	**< 0.001**
CTP	9 (2.25)	9.5 (3.0)	10.5 (3.5)	10 (2.5)	8.5 (2.25)	0.111	0.870	**< 0.001**	**0.003**
Novel urinary biomarkers									
Urinary NGAL (pg/mL)	159 (13)	217.15 (206.97)	422.85 (499.85)	189.85 (148.02)	211.25 (189.07)	**< 0.001**	**0.001**	0.778	**< 0.001**
Urinary IL-18 (pg/mL)	23.8 (18.83)	44.75 (34.7)	71.13 (65.45)	44.75 (25.53)	28.95 (31.78)	**< 0.001**	**0.001**	**0.012**	**< 0.001**
Urinary KIM-1 (ng/mL)	0.40 (0.49)	0.87 (1.18)	1.55 (3.91)	0.96 (1.57)	0.52 (0.81)	**< 0.001**	0.109	0.132	**0.004**

Data represented as *n* (%), *N = 100, unless otherwise specified, *ATN* acute tubular necrosis, *CTP* Child-Turcotte Pugh, *HRS* Hepatorenal syndrome, *IL-18* interleukin-18, *INR* International normalized ratio, *KIM-1* kidney injury molecule-1, *MASH* Metabolic Associated Steatohepatitis, *MELD-Na* Model for end-stage liver disease Sodium, *NGAL* neutrophil gelatinase-associated lipocalin, *PRA* prerenal azotemia, *SBP* spontaneous bacterial peritonitis *UGI* upper gastrointestinal

The bold values indicate statistical significance (*p*<0.05)

Serum creatinine was significantly different between cases (1.15 [1.17] mg/dl) and controls (1.85 [1.0] mg/dl), but could not distinguish between ATN and HRS or HRS and Pre-Renal AKI. Serum bilirubin was markedly raised in ATN patients (12.95 [19.27] mg/dL), followed by HRS (3.95 [9.98] mg/dL) and PRA (2.25 [1.53] mg/dL). Both MELD-Na and CTP scores were significantly higher in cases as compared to controls and in ATN as compared to other causes of AKI (Table 1).

### Urinary biomarkers

Urinary NGAL was markedly elevated in ATN (422.85 [499.85] pg/mL) compared to both HRS (189.85 [148.02] pg/mL, *p* = 0.001) and PRA (211.25 [189.07] pg/mL, *p* < 0.001). However, NGAL values were not significantly different between HRS and PRA (*p* = 0.778). IL-18 values were the highest in ATN (71.13 [65.45] pg/mL), followed by HRS (44.75 [25.53] pg/mL) and PRA (28.95 [31.78] pg/mL), successfully differentiating ATN from both PRA (*p* < 0.001) and HRS (*p* = 0.012). IL-18 also effectively distinguished HRS from PRA (*p* = 0.008), indicating a central role in identifying varying degrees of tubular injury. In contrast, KIM-1 could only distinguish ATN (1.55 [3.91] ng/mL) from PRA (0.52 [0.81] ng/mL, *p* = 0.004). All three urinary biomarkers were significantly higher in cases than in controls (Table 1). A supplementary table S1 has been attached for reporting number of true positives (ATN correctly identified) and false positives (HRS/PRA mislabeled as ATN).

### ATN and Non-ATN groups (HRS and PRA patients)

Comparison between ATN and non-ATN (HRS and PRA) cases revealed serum creatinine to be modestly higher in ATN [2.3 (3.03) mg/dL vs. 1.75 (0.88) mg/dL; *p* = 0.01]. However, urinary NGAL [422.85 (I499.85) vs. 204 (159.35) pg/mL; *p* < 0.001] and IL-18 [71.13 (65.45) vs. 39.85 (34.08) pg/mL; *p* < 0.001] showed much stronger differentiation between these groups. Although KIM-1 was higher in ATN (1.55 [3.91] ng/mL) than in non-ATN (0.73 [0.99] ng/mL, *p* = 0.011), the *p*-value was comparable to that of serum creatinine (Table 2).

**Table 2:** Comparison of parameters between ATN and Non-ATN groups (HRS and PRA patients)

Parameters	ATN (*n* = 10)	Non ATN [HRS (*n* = 18), PRA (*n* = 22)]	*p*-value
Serum creatinine (mg/dL)	2.3 (3.03)	1.75 (0.88)	0.01
MELD Na (mEq/L)	30.5 (11.25)	22.5 (6.75)	< 0.001
CTP	10.5 (3.5)	9 (2.75)	0.131
NGAL (pg/mL)	422.85 (499.85)	204 (159.35)	< 0.001
IL-18 (pg/mL)	71.13 (65.45)	39.85 (34.08)	< 0.001
KIM-1 (ng/mL)	1.55 (3.91)	0.73 (0.99)	0.011

*ATN* acute tubular necrosis, *CTP* Child-Turcotte Pugh, *HRS* Hepatorenal syndrome, *IL-18* interleukin-18, *KIM-1* Kidney Injury Molecule-1, *MELD-Na* Model for end-stage liver disease Sodium, *NGAL* neutrophil gelatinase-associated lipocalin, *PRA* prerenal azotemia

### Diagnostic accuracy

#### ATN vs. HRS

**Fig. 1:**
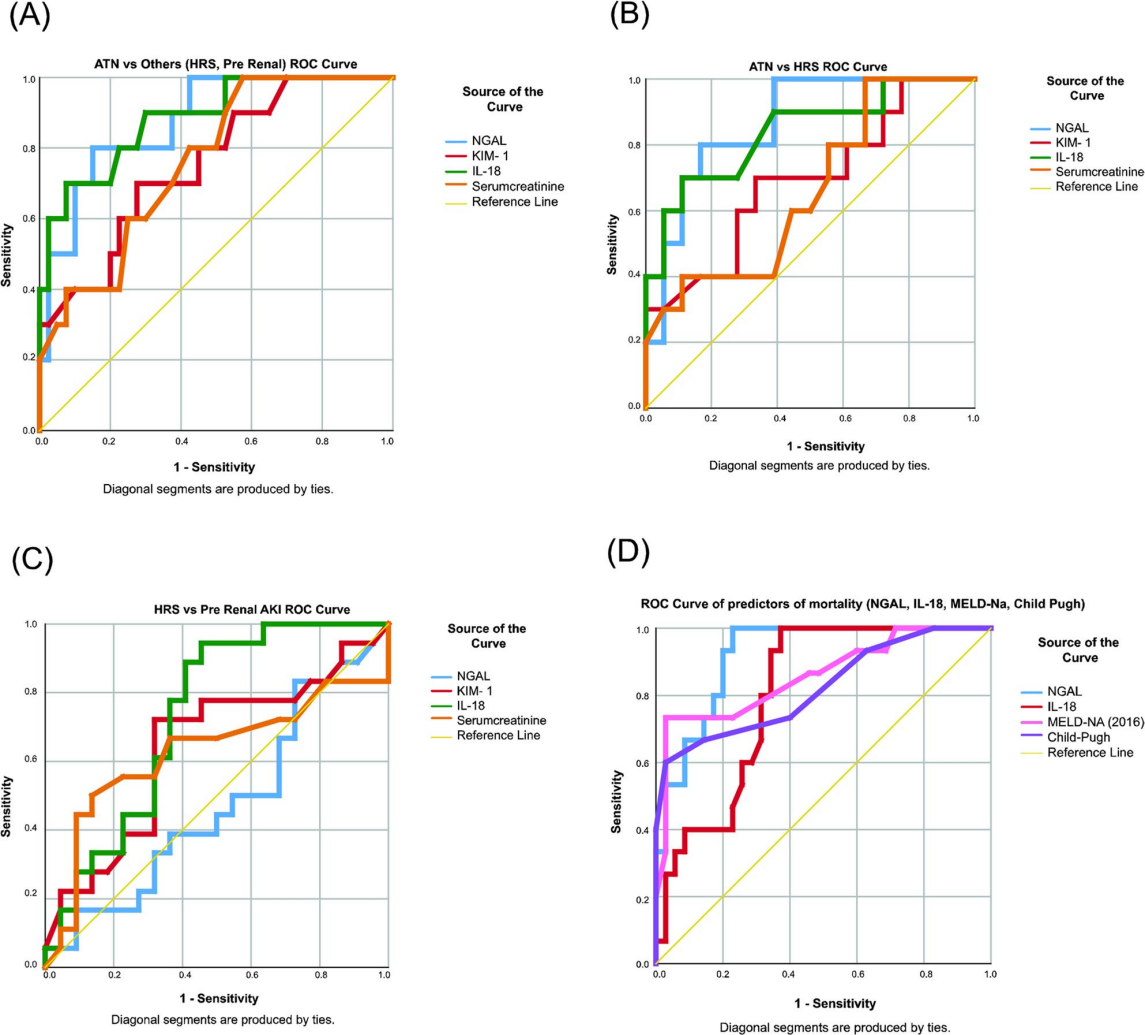
ROC curves of a. ATN vs others b. ATN vs HRS c. HRS vs Pre-Renal AKI d. Predictors of mortality (NGAL, IL-18, MELD-Na, CTP)

ROC analysis (Table 3; Fig. 1b) demonstrated that NGAL, at a cutoff of 339.6 pg/mL, yielded an AUROC of 0.867, with 80% sensitivity and 83.3% specificity. IL-18 performed similarly (AUROC 0.839, cutoff 67.75 pg/mL; 70% sensitivity and 88.9% specificity), while KIM-1 and serum creatinine had inferior performance (AUROC 0.689 and 0.661, respectively).

**Table 3:** ROC Analysis of novel urinary biomarkers and serum creatinine in ATN vs HRS, ATN vs Non-ATN, HRS vs PRA, and predictors of mortality

Biomarkers	Cutoff	AUROC	95% C. I	Sensitivity (%)	Specificity (%)	Positive predictive power	Negative predictive power
ATN (*n*=10) vs HRS (*n*=18)							
NGAL (pg/mL)	339.6	0.867	0.733 to 1.000	80	83.3	0.727	0.882
IL-18 (pg/mL)	67.75	0.839	0.677 to 1.000	70	88.9	0.778	0.842
KIM-1 (ng/mL)	1.39	0.689	0.477 to 0.901	70	66.7	0.538	0.800
Serum Creatinine (mg/dL)	1.55	0.661	0.450 to 0.873	100	33.3	0.454	1.000
ATN (*n*=10) vs Non ATN [HRS (*n*=18), PRA (*n*=22)]							
NGAL (pg/mL)	358.15	0.878	0.768 to 0.987	80	85	0.571	0.944
IL-18 (pg/mL)	67.75	0.885	0.770 to 1.000	70	92.5	0.700	0.925
KIM-1 (ng/mL)	1.39	0.757	0.595 to 0.902	70	72.5	0.388	0.906
Serum Creatinine (mg/dL)	1.55	0.763	0.613 to 0.912	100	42.5	0.303	1.000
HRS (*n*=18) vs PRA (*n*=22)							
NGAL (pg/mL)	160.3	0.473	0.290 to 0.657	83.3	27.3	0.483	0.667
IL-18 (pg/mL)	30.95	0.732	0.576 to 0.889	94.4	54.5	0.629	0.923
KIM-1 (ng/mL)	0.73	0.641	0.463 to 0.820	72.2	68.2	0.650	0.750
Serum Creatinine (mg/dL)	2.05	0.625	0.435 to 0.815	50	86.4	0.750	0.678
Predictors of mortality							
NGAL (pg/mL)	266.5	0.920	0.848 to 0.992	100	77.1	0.652	1.00
IL-18 (pg/mL)	41.95	0.804	0.685 to 0.923	100	62.9	0.535	1.00
MELD-Na	29	0.858	0.735 to 0.981	73.3	97.1	0.916	0.894
CTP	11.5	0.822	0.684 to 0.960	60	97.1	0.900	0.85

*ATN* acute tubular necrosis, *C.I.* Confidence interval, *CTP* Child-Turcotte Pugh, *HRS* Hepatorenal syndrome, *IL-18* interleukin-18, *KIM-1 *kidney injury molecule-1, *MELD-Na* Model for End-Stage Liver Disease Sodium, *NGAL* neutrophil gelatinase-associated lipocalin, *PRA* prerenal azotemia

#### ATN vs. Non-ATN (HRS and PRA)

As shown in Table 3; Fig. 1a, NGAL at a cutoff of 358.15 pg/mL distinguished ATN from non-ATN cases with an AUROC of 0.878 (80% sensitivity, 85% specificity). IL-18 exhibited even higher diagnostic accuracy (AUROC 0.885, cutoff 67.75 pg/mL; 70% sensitivity and 92.5% specificity). KIM-1 and serum creatinine were less reliable in this differentiation. A pre-test clinical probability of ATN of 50% increased to 84.2% after a positive NGAL test and decreased to 19% after a negative NGAL test. Similarly, a positive IL-18 test raised the probability to 90.3%, and a negative test lowered it to 24.5%. Thus, both these biomarkers can vastly improve clinical decision-making while ruling in or ruling out ATN (Table 4).

**Table 4:** Comparison of pre-test and post-test probability of ATN for positive and negative tests of NGAL, IL-18, and KIM-1

Pre-test Probability of ATN (%)	Post-test probability of ATN after positive test (%)			Post-test probability of ATN after negative test (%)		
	NGAL (Positive)	IL-18 (Positive)	KIM-1 (Positive)	NGAL (Negative)	IL-18 (Negative)	KIM-1 (Negative)
10	37.2	50.9	22.0	2.5	3.5	4.4
30	69.6	80.0	52.2	9.2	12.2	15.1
50	84.2	90.3	71.8	19.0	24.5	29.3
70	92.6	95.6	85.6	35.4	43.1	49.1
90	98.0	98.8	95.8	67.9	74.5	78.8

#### HRS vs. PRA

In differentiating HRS from PRA (Table 3; Fig. 1c), NGAL was suboptimal (AUROC 0.473). In contrast, IL-18, with a cutoff of 30.95 pg/mL, achieved an AUROC of 0.732 (94.4% sensitivity, 54.5% specificity) and was the only biomarker that reliably distinguished these subtypes.

Univariate analysis (Table 5) identified higher serum creatinine (*p* = 0.035), bilirubin (*p* = 0.002), INR (*p* = 0.012), patients with hepatic encephalopathy (*p* = 0.001) and prolonged hospital stay (*p* = 0.024), MELD-Na (*p* < 0.001), and Child-Pugh scores (*p* = 0.001) as significant predictors of mortality. Urinary biomarkers, IL-18 (*p* = 0.017) and NGAL (*p* = 0.001), were significantly elevated in non-survivors, while KIM-1 did not differ significantly (*p* = 0.099). Although mortality rates were highest in ATN (40%) and HRS (38.9%) compared to PRA (18.2%), the type of AKI did not independently predict mortality.

**Table 5:** Predictors of 90-day mortality in AKI patients with decompensated cirrhosis in univariate analysis

Parameters	Died in 90 days	Alive at 90 days	*p*-value
Age (years)	39 (22)	43 (20)	0.803
Serum creatinine (mg/dl)	2.6 (2.9)	1.7 (0.8)	**0.035**
Serum bilirubin (mg/dl)	13.5 (20.7)	2.7 (2.1)	**0.002**
INR	1.7 (0.9)	1.4 (0.5)	**0.012**
Serum Sodium (mmol/L)	130.0 (11.0)	132 (4.0)	0.932
Serum Albumin (g/dl)	2.8 (0.5)	2.7 (0.8)	0.113
Presence of ascites	13 (86.6)	28 (80.0)	0.576
Total leukocyte count (counts per µL)	9470 (14200)	9100 (8970)	0.289
SBP	6 (40)	7 (20)	0.147
Hepatic encephalopathy	9 (60)	4 (11.42)	**0.001**
UGI Bleed	2 (13.3)	9 (25.71)	0.341
MELD-Na (2016)	32 (10)	22 (6)	**< 0.001**
CTP	12 (4)	9 (2)	**0.001**
Duration of hospital stay (days)	11 (9)	9 (9)	**0.024**
Type of AKI			
ATN (*n* = 10)	4 (40%)	6 (60%)	0.218*
HRS (*n* = 18)	7 (38.88%)	11 (61.11%)	0.178**
PRA (*n* = 22)	4 (18.18%)	18 (81.81%)	-
NGAL (pg/mL)	397.3 (466.2)	189 (94.2)	**0.001**
KIM-1 (ng/mL)	0.88 (2.3)	0.86 (0.98)	0.099
IL-18 (pg/mL)	59 (30.9)	36.2 (35.3)	**0.017**

Data represented as *n* (%), *N* = 100, unless otherwise specified, ^*^ATN vs. PRA, ^**^HRS vs. PRA, ATN, acute tubular necrosis, *CTP* Child-Turcotte Pugh, *HRS* Hepatorenal syndrome, *IL-18* interleukin-18, *INR* International normalized ratio, *KIM-1 *kidney injury molecule-1, *MELD-Na* Model for end-stage liver disease Sodium, *NGAL* neutrophil gelatinase-associated lipocalin, *PRA* prerenal azotemia, *SBP* spontaneous bacterial peritonitis

The bold values indicate statistical significance (*p*<0.05)

Multivariate logistic regression adjusted for all factors found significant in univariate analysis (Model 1) revealed that only urinary NGAL remained a significant predictor of mortality, with each unit increase in NGAL associated with a 2.4% increase in the odds of death (AOR = 1.024, 95% CI: 1.005–1.043, *p* = 0.014). Since serum creatinine, bilirubin, and INR contribute to MELD-Na scores and hepatic encephalopathy is considered in CTP scores, Models 2 and 3 were constructed to avoid multicollinearity. Model 2, adjusted for hepatic encephalopathy, hospital stay, and MELD-Na, and Model 3, adjusted for serum creatinine, hospital stay, and Child-Pugh score, confirmed NGAL as an independent predictor (AOR = 1.017, 95% CI: 1.003–1.031, *p* = 0.015; AOR = 1.023, 95% CI: 1.007–1.039, *p* = 0.005, respectively).

In ROC analysis, NGAL (AUROC: 0.920) bettered MELD-Na (AUROC: 0.858) as the best predictor of short-term mortality with the highest sensitivity of 100%, and 77.1% specificity at a cutoff of 266.50 pg/mL. Child-Pugh score (AUROC 0.822) was highly specific (97.1%) but less sensitive (60%) (Table 3; Fig. 1 d).

## Discussion

Our study reinforces the inadequacy of serum creatinine in delineating the cause of AKI, as demonstrated by its modest diagnostic performance while discriminating among various AKI etiologies. In contrast, novel urinary biomarkers, particularly IL-18 and NGAL, provided more specific insights into the underlying cause of AKI - their levels were highest in ATN, where there is structural damage to the kidney. Several studies have documented a graded increase in NGAL from pre-renal AKI to HRS to ATN [18]. Urinary NGAL and IL-18 distinguished ATN from other causes of AKI with AUROCs of 0.878 and 0.885 which is comparable to the values obtained by Belcher et al. and the meta-analysis by Puthumana et al. NGAL values in our cohort of HRS and pre-renal AKI patients were similar, unlike those previous studies, which is attributable to our small sample size [19, 20]. The consistency across independent datasets supports the robustness of these markers despite our small sample size.

Notably, our NGAL cutoff for distinguishing ATN from other AKI causes (358.15 pg/mL) closely mirrors the number reported by Belcher et al. (365 pg/mL), while Ariza et al. arrived at a lower cutoff of 236 pg/mL. IL-18 showed a progressive rise from pre-renal AKI to ATN, confirming the theory of structural damage in HRS. Our IL-18 cutoff (67.75 pg/mL) was similar to the values published by Ariza et al. (54 pg/mL) and Belcher et al. (85 pg/mL) [20, 21].

The modest performance of KIM-1 was in line with the results of prior cirrhosis cohort. This may reflect differences in marker kinetics: NGAL and IL-18 are rapidly released in acute tubular injury, whereas KIM-1 elevation may be delayed or less pronounced in the setting of cirrhosis-related ATN.

The clinical utility of these biomarkers lies in their ability to stratify the cause of AKI at diagnosis, thereby streamlining treatment decisions. In clinical practice, differentiating HRS from ATN is useful as terlipressin therapy is effective only in the former. NGAL and IL-18 can raise or lower the post-test probability of ATN which can direct appropriate terlipressin therapy. These could also support the prompt identification of a kidney transplant candidate by detecting tubular damage. The inclusion of a sizable control group in our study, which was frequently absent from earlier research, enabled us to perform baseline comparisons.

The ability of infections, UTIs, to raise biomarker levels, particularly NGAL, remains a concern [11]. Although we excluded patients with UTI from our study, nosocomial UTIs pose a significant challenge in the real-world application of novel biomarkers for the diagnosis of AKI in DCLD patients in India, given the prolonged hospital stays and immunocompromised status of these patients [22].

NGAL in particular performed exceedingly well in predicting 90-day mortality among our cases. It maintained a robust association with mortality prediction across logistic regression models, outperformed MELD and CTP scores, and returned an AUROC value of 0.920 that compared favorably with previous studies (0.697 by Allegriti et al. and 0.876 by Ariza et al.) [18, 21]. Prognostic enrichment of such precision can guide timely ICU admission and transplant referral.

Our diagnoses of ATN and HRS were based on clinical criteria rather than histological confirmation, as kidney biopsy, the gold standard for identifying tubular injury, was not feasible in this setting. This limitation may have influenced the discriminatory performance of the biomarkers [20, 21].

The study’s limitations include its single-center design and small sample size, especially in the ATN subgroup which may affect the generalizability of our findings. Multiple subgroup analyses were executed on smaller sample sizes, which contributed to wide confidence intervals and increased the chances of Type 1 errors. This being a pilot study, the results should be interpreted as exploratory and hypothesis-generating rather than conclusive. Selection bias is possible despite consecutive enrollment. Cost-effectiveness analyses were not performed but the availability of automated assays and ELISA-based assays means scalability is achievable in the future. Given the dynamic nature of urinary biomarkers, serial measurements of urinary biomarker levels, as done by Huelin et al., would provide better guidelines about the optimal timing for testing [23]. Additionally, further studies should assess whether these biomarkers can predict AKI in DCLD patients with normal creatinine levels. Interestingly, bilirubin levels in ATN cases were significantly elevated, warranting further research into how bile acids contribute to tubular damage.

Our study lends further credence to the incorporation of NGAL and IL-18 into a multimodal diagnostic framework for early and accurate differentiation of AKI subtypes in DCLD, which may ultimately guide more tailored therapeutic interventions and improve patient outcomes. Furthermore, their strong predictive power in determining mortality might complement traditional clinical scores like MELD-Na in early risk stratification of patients and identifying patient groups who require closer monitoring and aggressive management. Future multicenter studies are needed to validate these findings and to establish precise cutoffs for diverse patient populations.

## Supplementary Information

Supplementary Material 1.

Supplementary Material 2.

## Data Availability

The datasets used and/or analysed during the current study are available from the corresponding author on reasonable request.
