# Supplementary material for: Novel urinary biomarkers to differentiate AKI etiologies and predict mortality in decompensated cirrhosis patients: a prospective cohort study
Source: BMC Gastroenterol. 2025 Dec 7;26:16. doi: 10.1186/s12876-025-04462-1 (PMC12784513; doi:10.1186/s12876-025-04462-1)
Supplement: Supplementary file 1 — Supplementary Material 1. [file 12876_2025_4462_MOESM1_ESM.docx]

QUESTIONNAIRE

1. PATIENT PARTICULARS
2. Hospital No.: MRD No. Bed No.

Date of admission: Date of Discharge: Duration of H. Stay:

1. Name:
2. Age: Sex: Religion/Caste:

Height: Weight:

1. Address:
2. Ph. No.
3. Category/Diagnosis:

Decompensated Cirrhosis: Decompensated Cirrhosis with evidence of azotemia:

1. Exclusion Criteria Checklist

Does the patient have any of the following?

1. History of Hepatorenal Syndrome – Non-AKI (formerly HRS Type II)

2. Prior liver transplant

3. Prior kidney transplant

4. Currently receiving or previously received renal replacement therapy (e.g., dialysis)

5. Evidence or history of urinary tract infection (UTI)

6. Known renal disease (e.g., glomerulonephritis, obstructive uropathy)

7. Congestive heart failure (NYHA Class > II)

8. Hepatocellular carcinoma (HCC)

9. Extrahepatic malignancy

10. Chronic obstructive pulmonary disease (COPD) — GOLD grade > II

11. Currently pregnant

12. Has the patient declined to give informed consent for the study?

1. Clinical History and Parameters
2. Vitals: a) BP: b) PR: c) RR: d) Temperature:
3. Any Comorbidities: Diabetes ______ Hypertension______
4. Any other significant medical/surgical history in past:
5. H/o Alcohol intake __________ (if yes, type; quantity) Smoking_________(pack yrs)
6. Diet: Veg/Non-Veg: Smoked food intake:
7. Pallor: Icterus: Edema:
8. Presence of Ascites:
9. Presence of Hepatic encephalopathy:
10. Evidence of Variceal bleed including Upper GI bleed:
11. Any recent h/o passage of black tarry stools/diarrhea/vomiting/haemorrhage/burn:
12. Any recent h/o fever, infections (Upper or Lower Respiratory Tract Infections) :
13. Urine:

a) Output/hr: b) Burning micturition: c) Blood in urine:

1. Any h/o cardiac diseases:
2. Any h/o Nephrotoxic Drug intake(mention drug):
3. Diuretic Challenge Test:
4. Etiology of Cirrhosis [Alcoholic/ MASH (Metabolic Dysfunction-associated Steatohepatitis)/Viral (Hepatitis B/C/ Others)/ Other causes (Wilson’s, Autoimmune causes etc.)]
5. Evidence of Spontaneous Bacterial peritonitis:
6. LABORATORY PARAMETERS:
7. Renal Function Test: **a)S. Creatinine:** Initial baseline:

Current levels (mention after how many hrs):

1. S. Na+: Urinary Na+ (if present):
2. S. Ammonia:
3. BUN (Blood Urea Nitrogen):
4. ACR (Albumin Creatinine Ratio):
5. Urine R/E:
6. Specific gravity:
7. Proteinuria/Glucosuria/Ketonuria:
8. Haematuria:
9. Pus cells:
10. Urinary Sediments and Casts:
11. Sepsis Screen (Mention Units wherever applicable)
12. Blood culture:
13. Total Leucocyte Count:
14. Differential Leucocyte count:
15. Platelet Count:
16. Hb%:
17. ESR:
18. CRP:
19. Pro-cal:
20. Serum Bilirubin:
21. S. Albumin:
22. INR:

4) Cardiac ECHO (if done):
