# Supplementary material for: Novel urinary biomarkers to differentiate AKI etiologies and predict mortality in decompensated cirrhosis patients: a prospective cohort study
Source: BMC Gastroenterol. 2025 Dec 7;26:16. doi: 10.1186/s12876-025-04462-1 (PMC12784513; doi:10.1186/s12876-025-04462-1)
Supplement: Supplementary file 2 — Supplementary Material 2. [file 12876_2025_4462_MOESM2_ESM.docx]

Supplementary Table S1. Diagnostic performance of urinary biomarkers for ATN at optimal ROC cut-offs

| Biomarkers | Cut off | True positives | False positives | True negatives | False Negatives |
| --- | --- | --- | --- | --- | --- |
| NGAL (pg/mL) | 358.15 | 8 | 6 | 34 | 2 |
| IL-18 (pg/mL) | 67.75 | 7 | 3 | 37 | 3 |
| KIM-1  (ng/mL) | 1.39 | 7 | 11 | 29 | 3 |
| Serum Creatinine (mg/dL) | 1.55 | 10 | 23 | 17 | 0 |
